# Giardia-filled Pancreatic Mass in a Patient With Recently Treated T-cell-rich B-cell Lymphoma

**DOI:** 10.7759/cureus.1019

**Published:** 2017-02-09

**Authors:** Raj Shah, Talal Asif, Richard Johnson

**Affiliations:** 1 Department of Internal Medicine, University of Missouri Kansas city; 2 Department of Internal Medicine, University of Missouri Kansas City; 3 Hematology and Oncology, University of Missouri Kansas City

**Keywords:** giardia, pancreatic mass

## Abstract

*Giardia lamblia* (*G. lamblia*)-filled pancreatic masses are a rarely reported entity. Furthermore, there are only a few case reports in literature on the association of these masses with cancer. We present a case of a *G. lamblia-*filled pancreatic cystic mass in a patient with a history of T-cell-rich B-cell lymphoma. The authors performed a PubMed search using (Medical Subject Headings) MeSH terms of pancreas, mass, *Giardia*, and lymphoma.

A 53-year-old male with past medical and surgical history of T-cell-rich B-cell lymphoma, status post R-CHOP (cyclophosphamide, doxorubicin, vincristine, and prednisone plus rituximab) therapy with positron emission tomography (PET) scan showing no residual disease, essential hypertension, and alcohol use disorder presented to the emergency department (ED) with epigastric pain and nausea for one week. Computed tomography (CT) scan of the abdomen showed a 2.3 cm hypodense pancreatic cystic mass. This was a new finding when compared to his prior abdominal imaging. Fine needle aspiration (FNA) biopsy of the mass showed lymphocytes, reactive atypical epithelial cells, and numerous organisms consistent with *Giardia lamblia*. He was treated with metronidazole 250 mg by mouth three times a day (TID) for five days. Follow-up magnetic resonance imaging (MRI) and magnetic resonance cholangiopancreatography (MRCP) showed complete resolution of the pancreatic mass.

There are only a few case reports on *G. lamblia *in the pancreas. The pathologist indicated sheets of numerous *Giardia* in the sample, making small bowel contamination less likely and *G. lamblia* aspirate from the pancreas more probable as the source. The authors hypothesize that this patient may have had chronic *G. lamblia *infection as a potential cause for the T-cell-rich B-cell lymphoma manifestation. The patient reported travel to an area with possible exposure to *G. lamblia* one year prior to presentation with the lymphoma. During that time he had increasing abdominal pain, intermittent chronic diarrhea, and weight loss. *G. lamblia’s* mechanism of action has been theorized to involve induction of pro-apoptotic factors, intestinal barrier dysfunction, up-regulation of cell-cycle genes, and crypt hyperplasia.

The mechanism of action of pancreatic masses filled with *G. lamblia* and the association of *G. lamblia* and cancer is not completely understood. Further research is required to better understand these possible phenomena as it can help us better comprehend *G. lamblia*, its associations, and new cancer etiologies.

## Introduction

*Giardia lamblia* (*G. lamblia*) is a flagellated binucleate protozoan parasite that commonly manifests with abdominal pain, nausea, diarrhea, or even asymptomatically ​​[1]. *G.lamblia-*filled pancreatic masses are a rarely reported entity. Furthermore, masses associated with cancer exist but only in a few case reports. We present a case of a *G. lamblia-*filled pancreatic cystic mass in a patient with a history of T-cell-rich B-cell lymphoma. Informed consent was obtained from the patient for this study.

## Case presentation

A 53-year-old male with a past medical and surgical history of T-cell-rich B-cell lymphoma, status post R-CHOP (cyclophosphamide, doxorubicin, vincristine, and prednisone plus rituximab) therapy with positron emission tomography (PET) scan showing no residual disease, essential hypertension, and alcohol use disorder presented to the emergency department (ED) with a one-week history of epigastric pain and nausea. The pain was sharp and stabbing in character, non-radiating, worsened by movement, relieved by bending forward, and 5/10 on a pain severity scale. He had associated nausea but no vomiting, diarrhea, or constipation.

On physical examination, the blood pressure was 139/84 mmHg, the pulse 104/min, the temperature 98˚F and the respiratory rate 14/min. An abdominal examination showed diffuse tenderness to deep palpation especially in the epigastrium. There was no guarding or rigidity. The bowel sounds were normal. The cardiovascular, respiratory, and neurological exams were otherwise unremarkable.

**Figure 1:**
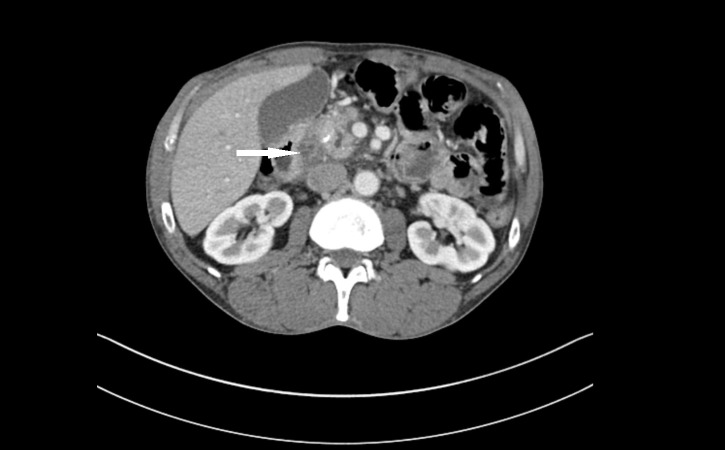
CT of the abdomen. Arrow shows a 2.3 cm hypodensity between the duodenum and the head of the pancreas, possibly representing a pancreatic neoplasm.

Routine labs including complete blood count, basic metabolic panel, liver panel, serum lipase, and alcohol level were within normal limits. A computed tomographic (CT) scan of the abdomen (Figure 1) showed a 2.3 centimeter (cm) hypodense cystic mass that could represent a pancreatic neoplasm between the duodenum and the head of the pancreas. This mass was a new finding when compared with the abdominal CT scan done one year prior at the time of diagnosis of his T-cell-rich B cell lymphoma.

**Figure 2:**
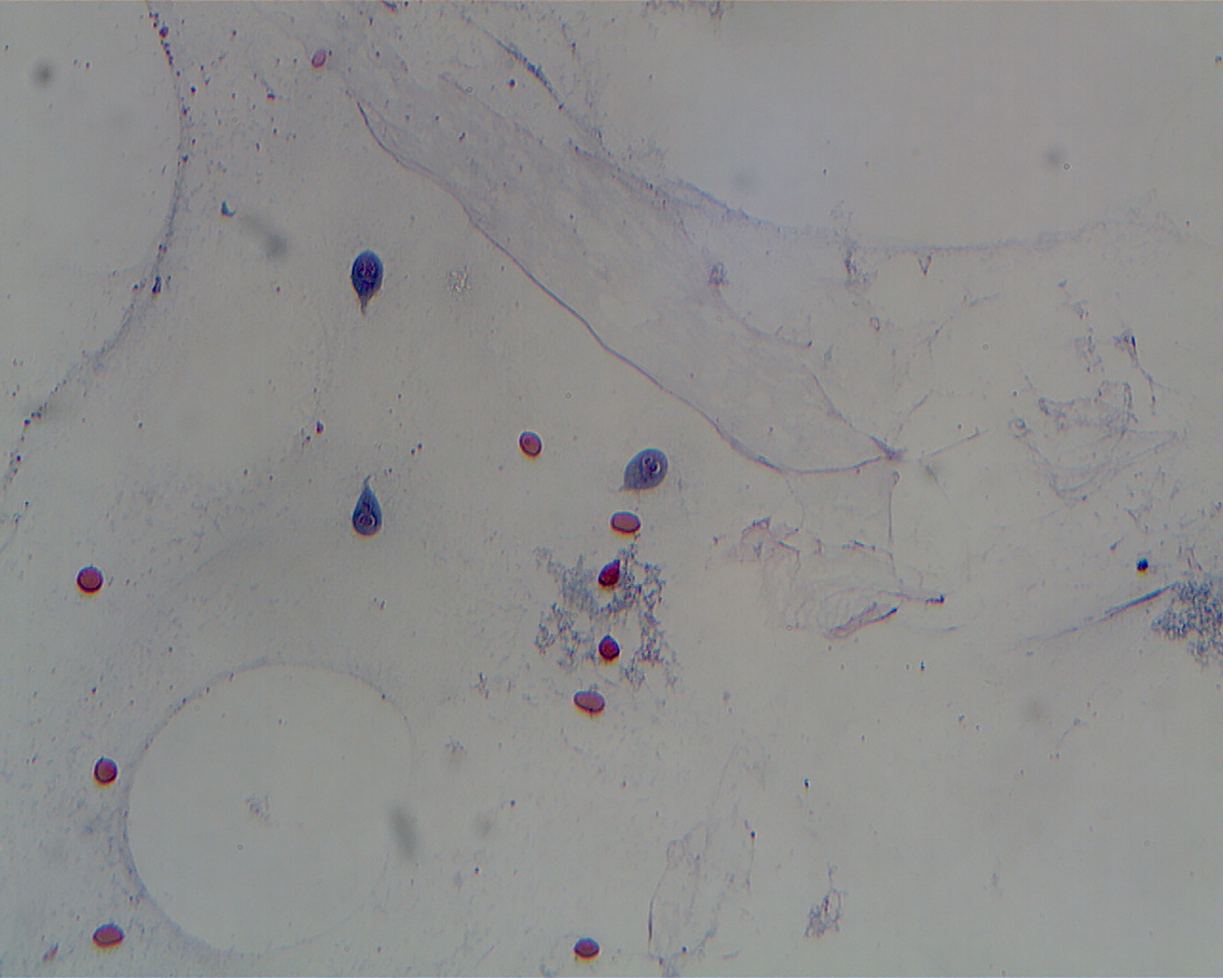
Slide of the fine needle aspirate showing Giardia Lamblia.

The patient underwent esophagogastroduodenoscopy (EGD) and endoscopic ultrasound (EUS) with fine needle aspiration (FNA) biopsy. The EGD showed edema and mucosal swelling in the duodenal bulb secondary to extrinsic compression. The EUS further characterized this as a 2.5 x 2.7 cm calcification in the head of the pancreas with hypoechogenecity causing a mass-like effect. A total of five passes using a 22 gauge needle FNA showed lymphocytes, reactive atypical epithelial cells, and numerous organisms consistent with *Giardia lamblia* (Figure 2). He was treated with metronidazole 250 mg by mouth (PO) three times a day (TID) for five days. On follow-up magnetic resonance imaging (MRI) and magnetic resonance cholangiopancreatography (MRCP), the pancreatic cystic mass had completely disappeared.​

## Discussion

Rare case reports have been published in literature on *Giardia lamblia* in the pancreas. Miyahara, et al. found *G. lamblia* via endoscopic retrograde cholangiopancreatography (ERCP) in a diabetic pancreas, which was treated with metronidazole restoring pancreatic exocrine function [2]. Carter, et al. reported a patient with abdominal pain and chronic intermittent diarrhea who was found to have a pancreatic mass filled with* G. lamblia* [3].

*Schistosoma hematobium*, *Opisthorchis,* and *Clonorchis* are all parasites well associated with carcinogenesis, but *G. lamblia* has not had a proven association although suspicion exists [4]. Nagasaki, et al. report a case of gallbladder cancer associated with *G. lamblia* in the bile juice [5]. Shirsat, et al. report a coexisting case of low grade duodenal MALT lymphoma (MALToma) and *G. lamblia* [6]. Misra, et al. looked at the association of gastric giardiasis and carcinoma stomach and found that eight of 54 biopsies of gastric carcinoma cases contained *G. lamblia* [7]. Pu, et al. report *G. lamblia* enteritis in a cecal mass aspirate [8]. The authors also hypothesize that this patient may have had chronic *G. lamblia* infection as a potential cause for the T-cell-rich B-cell lymphoma manifestation. The patient reported travel to an area with possible exposure to *G. lamblia* one year prior to presentation for the lymphoma. During that time he had increasing abdominal pain, intermittent chronic diarrhea and inability to keep weight on. *G. lamblia’s* mechanism of action has been hypothesized to involve induction of pro-apoptotic factors, intestinal barrier dysfunction, up-regulation of cell-cycle genes, and crypt hyperplasia [9]. Documented nodular lymphoid hyperplasia has been associated with *G. lamblia* [10]. Nodular lymphoid hyperplasia is considered a risk factor for lymphoma and thus may explain lymphoma development in susceptible individuals.

This parasite is characterized as an opportunistic infection, which makes it difficult to discern if the malignancy allowed *Giardia* to invade more easily or if *Giardia* had a role in the malignancy’s manifestation. Numerous confounding factors and limited previous reports make it difficult to ensure a true association.

Additionally, the patient had no recent history to point to a *G. lamblia* infection such as fecal oral contact, camping, or drinking from a contaminated water source. However, the chronicity of his overall symptoms and the distance of likely infection would more likely lead to a chronic inflammatory state. Furthermore, *Giardia* does not typically invade tissue and is usually found in the small bowel. The possibility of the EUS fine needle becoming contaminated via the *G. lamblia* in the small bowel tissue exists but the description by multiple physicians attested to “sheets” of *Giardia* as the most likely source. The pathologist-indicated sheets of numerous *Giardia* in the sample make the small bowel contamination less likely and *G. lamblia* aspirate from the pancreas more probable as the source. How the parasite invaded the pancreatic tissue remains unknown, but the Nagasaki, et al. case does suggest possible motility up the ducts.

## Conclusions

The mechanism of action of pancreatic masses filled with *G. lamblia* and the association of *G. lamblia* and cancer is not completely understood. Further research is required to help better understand these possible phenomena and help us better comprehend *G. lamblia*, its associations, and new cancer etiologies.

## References

[1] Halliez MC, Buret AG (2013). Extra-intestinal and long term consequences of giardia duodenalis infections. World J Gastroenterol.

[2] Miyahara T, Kubokawa M, Koyanagi S (1997). A case of successfully treated giardiasis in pancreas. Fukuoka Igaku Zasshi.

[3] Carter JE, Nelson JJ, Eves M ( 2007). Giardia lamblia infection diagnosed by endoscopic ultrasound‐guided fine‐needle aspiration. Diagn Cytopathol.

[4] Peterson MR, Weidner N (2011). Gastrointestinal neoplasia associated with bowel parasitosis: real or imaginary?. J Trop Med.

[5] Nagasaki T, Komatsu H, Shibata Y (2011). A rare case of gallbladder cancer with giardiasis. Nihon Shokakibyo Gakkai zasshi = Jpn J Gastroenterol.

[6] Shirsat HS, Vaiphei K (2014). Primary gastrointestinal lymphomas-a study of 81 cases from a tertiary healthcare centre. Indian J Cancer.

[7] Misra V, Misra SP, Dwivedi M (2006). Giardia lamblia trophozoites in gastric biopsies. Indian J Pathol Microbiol.

[8] Pu RT, Rosenthal DL (2005). Incidental giardiasis diagnosed by fine‐needle aspiration of a phantom cecal “mass”. Diagn Cytopathol.

[9] Halliez MC, Buret AG (2013). Extra-intestinal and long term consequences of giardia duodenalis infections. World J Gastroenterol.

[10] Baran B, Gulluoglu M, Akyuz F (2013). Nodular lymphoid hyperplasia of duodenum caused by giardiasis. Clin Gastroenterol Hepatol.

